# Short term fat feeding rapidly increases plasma insulin but does not result in dyslipidaemia

**DOI:** 10.3389/fphys.2014.00469

**Published:** 2014-12-02

**Authors:** Benjamin Barzel, Jacquelyn M. Weir, Peter J. Meikle, Sandra L. Burke, James A. Armitage, Geoffrey A. Head

**Affiliations:** ^1^Neuropharmacology Laboratory, BakerIDI Heart and Diabetes InstituteMelbourne, VIC, Australia; ^2^Department of Anatomy and Developmental Biology, Monash UniversityMelbourne, VIC, Australia; ^3^School of Medicine (Optometry), Deakin UniversityGeelong, VIC, Australia; ^4^Department of Pharmacology, Monash UniversityMelbourne, VIC, Australia

**Keywords:** insulin, leptin, plasma lipids, obesity, hypertension

## Abstract

Although the association between obesity and hypertension is well-known, the underlying mechanism remains elusive. Previously, we have shown that 3 week fat feeding in rabbits produces greater visceral adiposity, hypertension, tachycardia and elevated renal sympathetic nerve activity (RSNA) compared to rabbits on a normal diet. Because hyperinsulinaemia, hyperleptinemia, and dyslipidaemia are independent cardiovascular risk factors associated with hypertension we compared plasma insulin, leptin, and lipid profiles in male New Zealand White rabbits fed a normal fat diet (NFD 4.3% fat, *n* = 11) or high fat diet (HFD 13.4% fat, *n* = 13) at days 1, 2, 3 and weeks 1, 2, 3 of the diet. Plasma concentrations of diacylglyceride (DG), triacylglyceride (TG), ceramide and cholesteryl esters (CE) were obtained after analysis by liquid chromatography mass spectrometry. Plasma insulin and glucose increased within the first 3 days of the diet in HFD rabbits (*P* < 0.05) and remained elevated at week 1 (*P* < 0.05). Blood pressure and heart rate (HR) followed a similar pattern. By contrast, in both groups, plasma leptin levels remained unchanged during the first few days (*P* > 0.05), increasing by week 3 in fat fed animals alone (*P* < 0.05). Concentrations of total DG, TG, CE, and Ceramide at week 3 did not differ between groups (*P* > 0.05). Our data show plasma insulin increases rapidly following consumption of a HFD and suggests that it may play a role in the rapid rise of blood pressure. Dyslipidaemia does not appear to contribute to the hypertension in this animal model.

## Introduction

Obesity is associated with increased mean arterial pressure (MAP) and renal sympathetic nerve activity (RSNA). Accumulating evidence suggests these changes are due to greater circulating concentrations of the adipokine leptin (Burke et al., 2013; Lim et al., 2013) which strongly correlate with RSNA and MAP in animal models of obesity (Prior et al., 2010; Burke et al., 2013). Consumption of a high fat diet (HFD) augments MAP and heart rate (HR) within the first few days of the diet, prior to any change in bodyweight (Burke et al., 2013). However, levels of circulating leptin are proportional to adiposity (Considine et al., 1996) and only begin to increase by the end of the first week of a HFD (Armitage et al., 2012). Thus, rapid changes in cardiovascular parameters suggest that a separate, leptin independent mechanism initiates the pressor response to a HFD. Plasma insulin concentrations increase within hours of meal consumption (Cummings et al., 2001) and are greater in both obese animals and humans (Bagdade et al., 1967; Lim et al., 2013) as well as patients with essential hypertension (Sobotka et al., 2011). Importantly, insulin is known to signal at the arcuate nucleus of the hypothalamus, the same nucleus at which a multitude of peripheral signals, including leptin, act to regulate energy and haemodynamic homeostasis (Benoit et al., 2004). Central administration of insulin attenuates food intake (Air et al., 2002) and augments sympathetic output (Muntzel et al., 1994). We have previously shown that insulin signaling is one of the factors responsible for the development of obesity related hypertension which is later maintained by slowly rising circulating leptin concentrations (Lim et al., 2013).

The association between dyslipidaemia and obesity is important given several lipid species are associated with a number of cardiovascular risk factors (Siri-Tarino et al., 2010). In addition, a single high-fat meal has been shown to reduce endothelial-dependent vasodilation up to 4 h post consumption in healthy normotensive individuals (Vogel et al., 1997). It has been suggested that endothelial-mediated vasodilatory mechanisms are impaired by triacylglycerides (TG) and free fatty acids (Doi et al., 1998; Lundman et al., 2001). Thus, it is possible that diet-induced changes in lipid profiles may play an early role in the development of obesity related hypertension. Lipid profiles have received scant attention in the fat-fed rabbit model of obesity related hypertension and only after several weeks of fat feeding (Eppel et al., 2013). The contribution of dyslipidaemia to the progression of disease is well-documented. Increased circulating ceramide concentrations are known to increase in obesity and are inversely correlated with insulin resistance (Haus et al., 2009). In addition, circulating levels of TG and cholesteryl esters (CE) are also elevated in obesity and have been shown to affect fasting glucose and insulin sensitivity (Sassolas et al., 1981; Cameron et al., 2008). In the present study the effect of HFD consumption on plasma insulin, leptin, and plasma lipid profiles was assessed in order to elucidate the contribution of each to the rapid rise in MAP observed within the first week of the diet.

## Materials and methods

### Animals and diets

Experiments were approved by the Alfred Medical Research Education Precinct Animal Ethics Committee and conducted in accordance with the Australian Code of Practice for Scientific Use of Animals. Experiments were conducted in 24 conscious male New Zealand White rabbits (2.76–2.90 kg). Rabbits were housed in individual cages with a telemetry blood pressure receiver (model RLA 1020, Data Sciences International, St. Paul, MN, U.S.A) fitted to the door, under controlled light (6:00–18:00) and temperature (22°C ± 2°C) conditions. Rabbits were initially fed a restricted (150 g daily) normal-fat diet (NFD; 4.3 % total fat, 2.63 kcal/g, Specialty Feeds, Glen Forest, Australia) but after baseline recordings were randomized into two dietary groups and given free access to either a NFD or a high-fat diet (HFD; 13.4 % total fat, 3.34 kcal/g, Specialty Feeds) for 3 weeks. Daily food intake was determined by weighing the contents of the food hopper daily as well as weighing the food added.

### Experimental procedures

A subset of rabbits underwent a preliminary operation under isoflurane anesthesia (3–4% in 1L/min oxygen; Abbot, Botany, NSW, Australia) following induction with propofol (10 mg/kg, Fresenius Kabi, Pymble, NSW, Australia). A radiotelemetry transmitter (model TA11PA-D70, Data Sciences) and catheter was implanted in the aorta via a small branch of the left iliac artery. Carprofen (3 mg/kg, Pfizer, Noth Ryde, NSW, Australia) was given before and 24 h after surgery for analgesia. After 1 week recovery, baseline MAP and HR were measured in the laboratory both by telemetry and by a catheter in the central ear artery. The telemetry signal was calibrated to the ear artery signal and this adjustment was applied to MAP measured in the home cage to minimize the possibility of drift of the signal with time. Baseline home cage MAP and HR were recorded for 1–2 days before rabbits were allocated to a group to receive either NFD or HFD. Home cage measurements were made continuously over 2 weeks.

### Plasma collection and analysis

In order to avoid the effects of recent food consumption, animals were fasted for 4 h before blood samples were collected. Blood was collected before and on days 1, 2, 3, 7, 14, and 21 following the start of the HFD. Small samples of blood were used to measure blood glucose concentrations (Optium Xceed, Abbott, Doncaster, Victoria, Australia). Arterial blood (4 ml) was drawn into vacuum sealed cylinders containing K3EDTA (Vacuette Premium, Greiner bio-one, Wemmel, Belgium) and spun at 4°C for 10 min at 3000 RPM. Plasma aliquots (100 μl) were snap frozen in liquid nitrogen and stored at −80°C until use. Plasma lipid species were extracted into chloroform/methanol and quantified using high performance liquid chromatography-tandem mass spectrometry (Weir et al., 2013). Lipid species identified were diacylglycerides (DG), TG, ceramides (Cer), and CE. Total lipids within each class were calculated from the sum of the individual species. Plasma insulin and leptin concentrations were assessed using an ultra-sensitive insulin ELISA kit (Crystal Chem, Chicago, USA) with rabbit insulin standard and a radio immunoassay multispecies kit (LINCO Research, St Charles, MO, USA), respectively.

### Data analysis

MAP and HR, derived from the pressure pulse, were digitized online at 500 Hz using an analog-to-digital data acquisition card (National Instruments 6024E, Austin, Texas, USA) and averaged over 2 s. MAP and HR were collected continuously over each 24 h period and averaged over one hourly intervals. Data were analyzed by split-plot repeated measures ANOVA allowing for between and within animal comparisons using excel version 2010 (Microsoft). MAP and HR were analyzed by repeated measures analysis of variance that allowed for within-animal contrasts. Data collected at a single time point were analyzed using a One-Way ANOVA. Bonferroni corrections were used to control for Type 1 error. A two sided probability of *P* < 0.05 was considered significant. For all statistics shown we refer to the main effect as a subscript, e.g., *P*_baseline_ pertains to comparisons between groups made prior to the consumption of either diet, *P*_group_, refers to comparisons between HFD and NFD-fed rabbits during dietary intervention, *P*_diet_ refers to contrasts between baseline and dietary intervention within both NFD and HFD groups, *P*_time_, refers to comparisons within each group made between baseline and week 3 time points, *P*_dietxtime_ pertains to the interaction between diet and time.

## Results

### Effect of 3 week fat feeding on plasma insulin, glucose and leptin, food intake and haemodynamics

**Figure 1:**
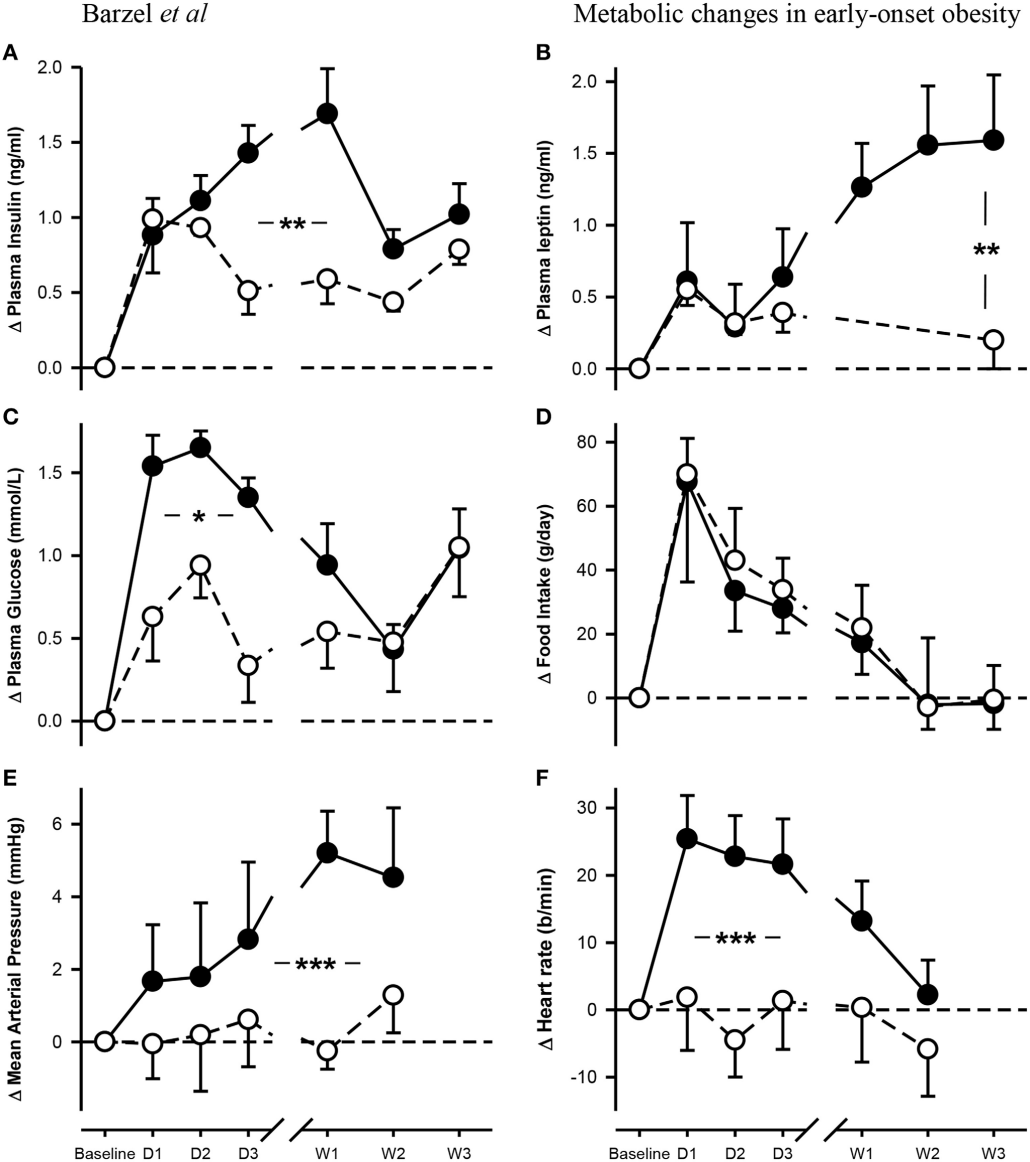
**Changes from baseline in levels of plasma insulin (A), leptin (B) and glucose (C) concentrations, food intake (D), mean arterial pressure (E) and heart rate (F) in rabbits fed either a normal fat diet (open circles) or a high-fat diet (closed circles) for 3 weeks**. Data are mean difference ± SED, ^*^P < 0.05, ^**^P < 0.01, ^***^P < 0.001 for differences between dietary groups. Day, D; Week, W.

Baseline plasma insulin concentrations were not different between the dietary groups and averaged 0.46 ± 0.03 ng/ml (*P*_baseline_ > 0.05; Figure 1, Table 1). A 50% increase from baseline in plasma insulin was observed in both NFD and HFD rabbits over the first 2 days of the diet (*P*_diet_ < 0.05 for both groups; Figure 1). A further increase in plasma insulin concentrations on day 3 resulted in 65% greater insulin concentrations in HFD compared with NFD animals at both day 3 and week 1 time points (*P*_group_ < 0.05; Figure 1). By week 2, insulin concentrations in HFD rabbits had decreased to those observed in NFD rabbits (*P*_group_ > 0.05; Figure 1). Plasma glucose concentrations at baseline were not different between the dietary groups and averaged 5.5 ± 0.12 mmol/L (*P*_baseline_ > 0.05; Figure 1, Table 1). Plasma glucose concentrations followed a similar pattern to insulin, rising on days 1 and 2 of the diet in both NFD and HFD rabbits (*P*_diet_ < 0.05 for both groups; Figure 1). However, HFD rabbits exhibited a 59% greater increase in plasma glucose concentrations than controls (*P*_group_ < 0.05). By week 2, glucose concentrations returned to levels observed in NFD rabbits (*P*_group_ > 0.05; Figure 1). By contrast, plasma leptin concentrations, which were averaged 0.91 ± 0.13 ng/ml at baseline (*P*_baseline_ > 0.05; Figure 1, Table 1), remained unchanged over the first 3 days of the diet in both dietary group (*P*_diet_ > 0.05; Figure 1). However, plasma leptin concentrations in HFD-fed rabbits increased on week 1 of the diet compared with baseline (*P*_diet_ > 0.05; Figure 1) and were 60 % greater than controls by the end of week 3 (*P*_group_ < 0.05; Figure 1). Food intake was similar in both groups with rabbits consuming 47–51% more food on the first day of both diets (*P*_diet_ < 0.05). Intake in both groups gradually diminished to baseline levels after the first week (Figure 1). HR also increased rapidly on the first day after the start of the HFD to a level 12% greater than baseline (*P*_diet_ < 0.001; Figure 1). HR remained elevated for the first week but had returned to control levels by week 2 (*P*_diet_ > 0.05). By contrast, MAP increased from baseline levels by the 3rd day of the HFD (*P*_diet_ < 0.05; Figure 1) and remained 7–8% elevated at 1–2 weeks (*P*_diet_ < 0.01; Figure 1). Both MAP and HR in HFD fed rabbits were markedly higher over the 2 weeks of measurements than those fed a NFD (*P*_group_ < 0.001; Figure 1).

**Table 1:** **Baseline concentrations of insulin, glucose, and leptin**.

	**Pre-NFD**	**Pre-HFD**	***P*_**group**_**
Insulin (ng/ml)	0.440 ± 0.036	0.472 ± 0.048	0.61
Glucose (mmol/l)	5.54 ± 0.20	5.42 ± 0.16	0.65
Leptin (ng/ml)	0.751 ± 0.058	0.964 ± 0.146	0.20

Values are mean ± SEM. P_group_ is comparison of normal fat diet (NFD) with high fat diet (HFD).

### Effect of HFD feeding on plasma lipid profiles

**Figure 2:**
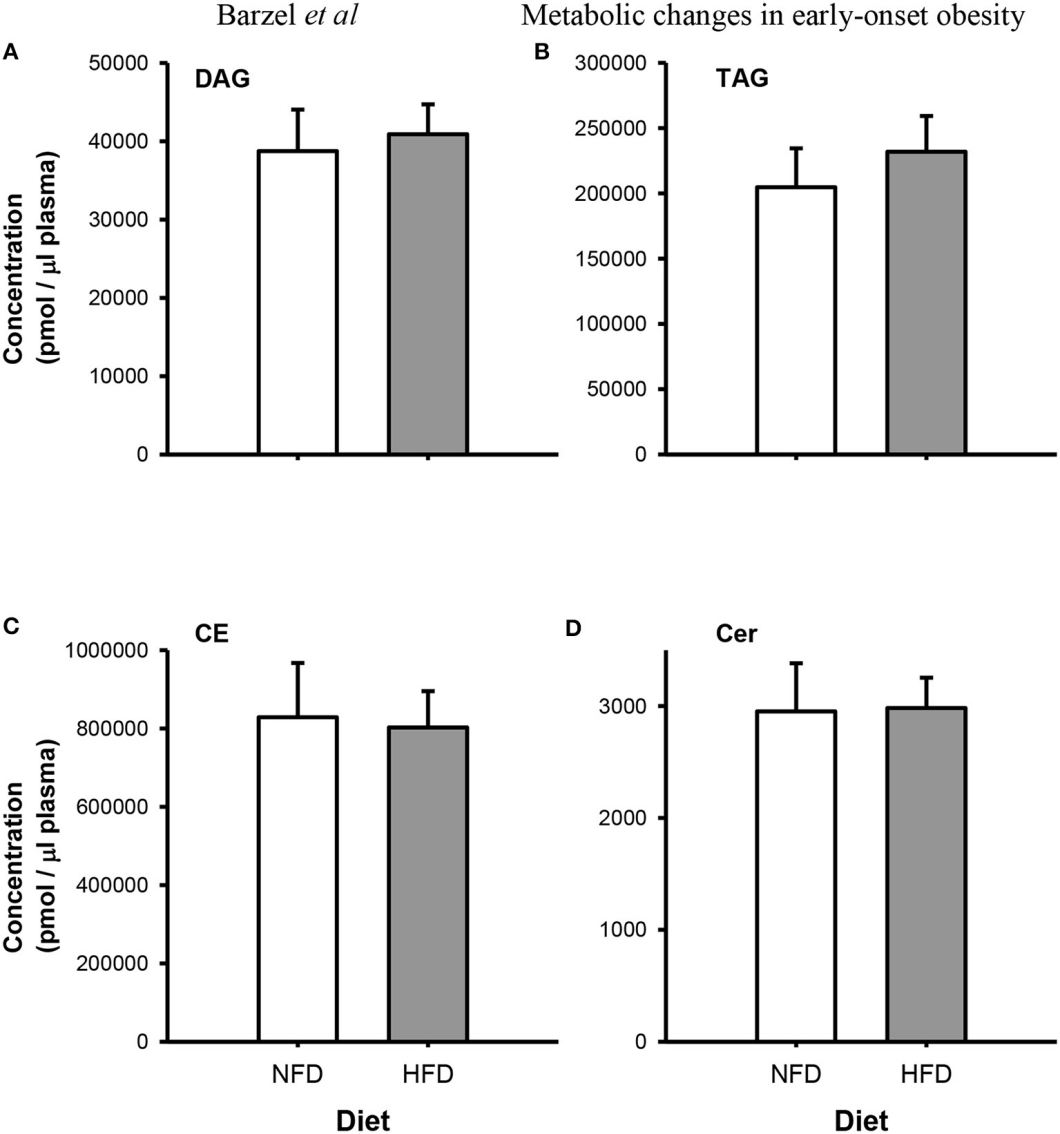
**Total Plasma concentrations of diacylglycerides (DG; A), triacylglycerides (TG; B), cholesteryl esters (CE; C) and ceramides (Cer; D) species in normal fat-fed (NFD; white bars) and high fat diet-fed (HFD; gray bars) after 3 weeks of diet**. Data are mean ± SEM.

After 3 weeks of diet, total plasma DG, TG, Cer, and CE concentrations were not different between the dietary groups (*P*_group_ > 0.05; Figure 2). Specific DG, TG, and CE species did not change over the 3-week diet in either dietary group (*P*_time_ > 0.05 for both NFD and HFD, Tables 2–5). By contrast, plasma Cer 16:0, 20:0, and 22:0 concentrations increased in HFD-fed rabbits over the 3 week period (*P*_time_ > 0.05; Table 2) yet this was unlikely due to the consumption of the HFD (*P*_diet_ > 0.05; Table 2) as the overall interaction between diet and time did not reach statistical significance (*P*_diet× time_ > 0.05; Table 2). Individual cholesteryl ester species at week 3 were not different between the dietary groups (*P*_group_ > 0.05; Table 3). Similarly, DG (*P*_group_ > 0.05; Table 4) and TG (*P*_group_ > 0.05; Table 5) lipid species were not different between the dietary groups.

**Table 2:** **Ceramide species at baseline and week 3 in both NFD and HFD–fed rabbits**.

	**NFD Week 0**		**NFD Week 3**		**HFD Week 0**		**HFD Week 3**		***P*_**diet**_**	***P*_**time**_**	***P*_diet×time_**
***n***	**9**		**10**		**10**		**12**				
**Ceramide species**	**Mean**	***SE***	**Mean**	***SE***	**Mean**	***SE***	**Mean**	***SE***			
Cer 16:0	189	28	253	28	189	16	287	21	1	0.01	1
Cer 18:0	131	18	136	17	139	13	179	26	1	1	1
Cer 20:0	168	22	206	27	159	12	239	21	1	0.05	1
Cer 22:0	608	93	754	108	550	47	882	84	1	0.05	1
Cer 24:1	440	71	633	94	395	43	510	50	1	0.21	1
Cer 24:0	833	141	971	174	665	56	885	95	1	1	1
Total Cer	2368	361	2952	430	2098	172	2983	271	1	0.18	1

Cer, Ceramides; NFD, normal fat diet; HFD, high fat diet.

**Table 3:** **Cholesteryl esters at baseline and week 3 in both NFD and HFD–fed rabbits**.

	**NFD Week 0**		**NFD Week 3**		**HFD Week 0**		**HFD Week 3**		***P*_**diet**_**	***P*_**time**_**	***P*_diet×time_**
***n***	**9**		**10**		**11**		**13**				
**Cholesteryl esters**	**Mean**	***SE***	**Mean**	***SE***	**Mean**	***SE***	**Mean**	***SE***			
CE 14:0	7697	1061	8329	1187	6480	1096	5407	453	0.73	1	1
CE 15:0	14345	2862	11136	2272	10088	2064	6288	946	0.63	1	1
CE 16:2	481	102	517	101	344	86	621	70	1	1	1
CE 16:1	36966	6848	56509	14258	28942	6014	30401	4080	0.97	1	1
CE 16:0	166404	29325	153289	29209	127649	25054	134902	19118	1	1	1
CE 17:1	9896	1933	6440	1188	7643	1094	5736	805	1	0.95	1
CE 17:0	11718	2693	6294	1127	8364	1989	5420	892	1	0.50	1
CE 18:3	17329	3009	20249	5470	13419	2731	21319	3599	1	1	1
CE 18:2	253823	46115	224743	32202	197578	35925	273220	36054	1	1	1
CE 18:1	154389	27782	154154	30973	96569	19247	121237	14200	1	1	1
CE 18:0	22633	5433	12713	2946	14992	4003	13617	2003	1	1	1
CE 20:5	894	257	1211	327	946	221	1382	258	1	1	1
CE 20:3	1113	229	1374	238	805	153	1182	168	1	1	1
CE 20:4	24310	5865	21934	3782	17486	3941	24641	3141	1	1	1
CE 20:2	204	38	244	51	214	55	239	42	1	1	1
CE 20:1	367	73	444	120	3211	2937	289	43	1	1	1
CE 20:0	477	89	308	62	1812	1449	259	46	1	1	1
CE 22:5	901	212	1116	339	2859	2087	1227	235	1	1	1
CE 22:4	293	79	280	63	256	62	262	37	1	1	1
CE 22:1	91	22	117	30	76	26	80	14	1	1	1
CE 22:0	221	32	177	36	372	210	144	25	1	1	1
CE 24:0	171	40	90	20	312	175	137	27	1	1	1
COH	125399	20715	147173	25523	98990	17953	154609	16540	1	1	1
Total CE	849914	142086	828575	138733	639050	113456	802445	92730	1	1	1

CE, cholesteryl esters; NFD, normal fat diet; HFD, high fat diet.

**Table 4:** **Diacylglycerides at baseline and week 3 in both NFD and HFD –fed rabbits**.

	**NFD Week 0**		**NFD Week 3**		**HFD Week 0**		**HFD Week 3**		***P*_**time**_**	***P*_**time**_**	***P*_diet×time_**
***n***	**9**		**10**		**11**		**13**				
**DG Species**	**Mean**	***SE***	**Mean**	***SE***	**Mean**	***SE***	**Mean**	***SE***			
DG 14:0 14:0	28	4	34	5	23	4	25	5	1	1	1
DG 14:0 16:0	378	52	448	54	343	47	375	42	1	1	1
DG 14:1 16:0	57	9	109	11	74	10	68	14	1	1	0.61
DG 16:0 16:0	1720	221	1753	201	1439	129	1689	213	1	1	1
DG 14:0 18:1	670	139	888	121	632	113	618	72	1	1	1
DG 14:0 18:2	436	70	402	80	386	59	518	46	1	1	1
DG 16:0 18:0	993	110	871	109	837	81	1024	101	1	1	1
DG 16:0 18:1	7054	1323	7679	932	6111	717	6785	739	1	1	1
DG 16:0 18:2	5986	877	4836	976	4203	629	7382	999	1	1	0.36
DG 16:1 18:1	1214	203	2012	227	1641	513	1223	147	1	1	1
DG 18:0 18:0	212	16	185	42	277	119	255	23	1	1	1
DG 18:0 18:1	1425	187	1444	151	1123	175	1384	110	1	1	1
DG 18:0 18:2	1184	143	1001	189	895	114	1431	145	1	1	0.35
DG 18:1 18:1	5021	767	6195	749	4079	691	4460	384	1	1	1
DG 16:0 20:3	90	16	92	12	201	118	97	14	1	1	1
DG 18:1 18:2	7275	1046	7253	1480	6040	711	8615	771	1	1	1
DG 16:0 20:4	156	19	112	21	123	15	198	36	1	1	0.49
DG 18:1 18:3	1069	159	1112	234	1648	767	1262	110	1	1	1
DG 18:2 18:2	1702	253	1670	481	1274	193	2647	302	1	0.62	0.45
DG 18:0 20:4	197	112	84	8	202	125	105	14	1	1	1
DG 18:1 20:3	184	28	171	26	347	223	164	18	1	1	1
DG 16:0 22:5	130	17	83	15	76	18	104	20	1	1	1
DG 18:1 20:4	374	59	288	49	270	37	425	59	1	1	0.56
DG 16:0 22:6	29	4	18	4	34	12	28	5	1	1	1
Total DG	37583	5289	38739	5294	32277	3603	40884	3828	1	1	1

DG, diacylglycerides, NFD, normal fat diet, HFD, high fat diet.

**Table 5:** **Triacylglycerides at baseline and week 3 in both NFD and HFD–fed rabbits**.

	**NFD Week 0**		**NFD Week 3**		**HFD Week 0**		**HFD Week 3**		***P*_diet_**	***P*_time_**	***P*_diet×time_**
***n***	**9**		**10**		**11**		**13**				
**TG Species**	**Mean**	***SE***	**Mean**	***SE***	**Mean**	***SE***	**Mean**	***SE***			
TG 14:0 16:0 18:2	3755	695	3729	521	2661	636	3812	495	1	1	1
TG 14:0 16:1 18:1	1644	426	3188	562	1728	463	1548	226	1	1	1
TG 14:0 16:1 18:2	432	94	557	74	585	202	600	76	1	1	1
TG 14:0 18:0 18:1	344	58	301	56	365	108	304	49	1	1	1
TG 14:0 18:2 18:2	514	90	493	114	729	313	767	110	1	1	1
TG 14:1 16:0 18:1	569	148	1139	249	742	196	584	130	1	1	1
TG 14:1 16:1 18:0	1798	450	3235	581	1729	375	1762	260	1	1	1
TG 14:1 18:0 18:2	117	35	303	54	4881	4747	193	30	1	1	1
TG 14:1 18:1 18:1	1378	299	1894	253	4834	3611	1644	186	1	1	1
TG 15:0 18:1 16:0	2032	209	1417	309	1809	419	1072	138	1	0.92	1
TG 15:0 18:1 18:1	1228	149	1075	216	2602	1586	754	93	1	1	1
TG 16:0 16:0 16:0	3150	560	2154	491	2434	591	3199	697	1	1	1
TG 16:0 16:0 18:0	2100	346	1811	675	1377	189	3107	616	1	1	1
TG 16:0 16:0 18:1	25852	3856	19383	3561	15841	3018	22531	3518	1	1	1
TG 16:0 16:0 18:2	12162	1992	7170	1940	7109	1269	15046	2945	1	1	0.23
TG 16:0 16:1 18:1	12109	2080	16866	3160	10526	2406	11433	1657	1	1	1
TG 16:0 18:0 18:1	7491	1216	4312	679	5718	807	5389	1143	1	1	1
TG 16:0 18:1 18:1	50498	5980	41240	5833	34880	7887	38074	4161	1	1	1
TG 16:0 18:1 18:2	35618	4652	23555	5079	24236	4482	36135	4229	1	1	0.56
TG 16:0 18:2 18:2	11604	1763	8206	2297	8113	1453	16218	2561	1	1	0.30
TG 16:1 16:1 16:1	173	41	284	39	291	130	191	25	1	1	1
TG 16:1 16:1 18:0	521	66	430	55	1047	639	528	73	1	1	1
TG 16:1 16:1 18:1	1723	299	1877	269	1293	302	1910	281	1	1	1
TG 16:1 18:1 18:1	2441	552	3941	619	2040	489	2530	313	1	1	1
TG 16:1 18:1 18:2	6301	1096	5597	980	5109	1078	7057	932	1	1	1
TG 17:0 16:0 16:1	4652	557	2903	503	3843	829	2066	300	1	0.15	1
TG 17:0 18:1 14:0	3653	450	2117	562	12151	9170	1141	203	1	1	1
TG 17:0 18:1 16:0	2101	257	1443	337	4914	3326	1402	192	1	1	1
TG 17:0 18:1 16:1	4237	499	3808	577	3463	941	2425	251	1	1	1
TG 17:0 18:1 18:1	2622	603	2397	375	2664	572	1902	440	1	1	1
TG 17:0 18:2 16:0	3291	423	2115	287	2559	532	1921	262	1	1	1
TG 18:0 18:0 18:0	71	26	31	7	1121	1084	55	11	1	1	1
TG 18:0 18:0 18:1	555	92	440	93	15842	15377	734	120	1	1	1
TG 18:0 18:1 18:1	5408	836	4963	817	31439	27087	6779	933	1	1	1
TG 18:0 18:2 18:2	1713	227	1334	352	4942	3562	2033	532	1	1	1
TG 18:1 14:0 16:0	4784	940	4858	781	3477	863	3857	641	1	1	1
TG 18:1 18:1 18:1	8080	1312	9679	1041	6304	1327	8604	873	1	1	1
TG 18:1 18:1 18:2	5822	917	6548	1358	4423	779	9515	1414	1	0.53	1
TG 18:1 18:1 20:4	345	67	1053	803	352	123	2421	763	1	0.82	1
TG 18:1 18:1 22:6	169	32	232	115	2570	2448	472	102	1	1	1
TG 18:1 18:2 18:2	4289	798	5209	1225	3480	616	7900	1428	1	0.68	1
TG 18:2 18:2 18:2	605	120	825	265	490	89	1440	341	1	0.72	1
TG 18:2 18:2 20:4	314	164	509	197	229	74	797	352	1	1	1
Total TG	238265	31727	204621	29850	246940	72879	231851	27372	1	1	1

TG, triacylglycerides, NFD, normal fat diet, HFD, high fat diet.

## Discussion

The main findings of the present study were that alongside elevations in blood pressure and HR, plasma glucose and insulin concentrations were increased within the first 3 days of a HFD, remaining elevated for the first week of the diet and returning to control levels thereafter. Notably, circulating leptin concentrations were unaltered by a HFD at day 3 but were markedly increased by week 3 whilst in the same time period, no evidence of dyslipidaemia was found. Together, these data suggest hyperinsulinemia rapidly develops after the commencement of a HFD and is a likely mechanism by which haemodynamics and sympathetic tone may change rapidly in the fat-fed rabbit model of obesity related hypertension.

A considerable body of evidence suggests insulin acts centrally to increase both blood pressure and sympathetic tone (Landsberg, 1996; Straznicky et al., 2010; Ward et al., 2011; Lim et al., 2013). There is a strong association between obesity, hyperinsulinemia and, at a later stage, insulin resistance (Weyer et al., 2001; Yuan et al., 2001). Of note is the apparent delay between the engagement of sympathetic nerve activity in obesity and the development of insulin resistance (Flaa et al., 2008) suggesting sympathetic overactivity may occur in response to very early changes in plasma insulin. Indeed central injections of insulin into the paraventricular nucleus of the hypothalamus produce large increases in lumbar sympathetic nerve activity (Ward et al., 2011). In the present study we observed a near two-fold increase in plasma glucose and insulin concentrations within 3 days of starting the HFD. Importantly, increases in MAP and HR in HFD-fed rabbits also began in the first few days of consumption as do increases in RSNA (Armitage et al., 2012; Burke et al., 2013) suggesting that circulating insulin may be involved in augmenting MAP early in the diet. In support of this are the findings that central administration of an insulin antagonist attenuated MAP after 1 week of a HFD (Lim et al., 2013). It is important to note that in the present study, plasma leptin concentrations in HFD-fed rabbits remained unchanged over the first 3 days of the diet but had increased by week 3. These results help explain our previous findings that central administration of a leptin antagonist to HFD-fed rabbits failed to elicit a reduction in either haemodynamic or sympathetic parameters at week 1 of the diet but produced large sympathoinhibitory and depressor responses at week 3 (Lim et al., 2013). Combined, these observations imply plasma insulin is involved in the remodeling of sympathetic tone within the first few days of consuming a HFD whilst leptin acts as a sympathoexcitatory signal later on in the diet, presumably once adiposity is increased. As both plasma glucose and insulin concentrations normalized by week 2 of the diet, the present observations point to sympathetic output preceding insulin resistance. Moreover, the apparent lack of effect of central administration of insulin on RSNA has been observed by others (Ward et al., 2011) and may in part be due to the direct effect of insulin on baroreflex gain (Pricher et al., 2008).

The present study also sought to establish the presence of dyslipidemia in our obese rabbit model and any subsequent contribution to the development of hypertension observed in these animals. In humans, dyslipidemia is a prominent feature of metabolic syndrome (Bays, 2009) and often appears in conjunction with hypertension (Nguyen et al., 2008). An example of the consequences of dyslipidemia can be found in greater total plasma ceramide concentrations which are known to occur in obesity whilst specific ceramide species are strongly associated with insulin resistance (Haus et al., 2009). In the present study, plasma concentrations of 4 lipid classes (Cer, CE, DG, and TG) were unchanged after 3 weeks of HFD. Our findings are in agreement with those made by Eppel and colleagues who observed no change in total plasma cholesteryl, and total plasma TG in rabbits fed a HFD for 9 weeks (Eppel et al., 2013) and suggests large changes in lipid profiles may take longer to develop in the rabbit model (Hamilton and Carroll, 1976). However, given the rapid haemodynamic and hormonal responses to dietary fat content, we expected to find changes in the expression of individual lipid species which would have been indicative of altered lipid metabolism. It is likely that our study was not powered to detect minute perturbations in the expression of specific plasma lipid species, contributing to our findings that plasma lipid profiles are unchanged by a diet high in fat. However, given that other parameters found in plasma, including insulin and leptin, can be measured accurately, our design is unlikely to be a confounding factor. Thus, our findings discount dyslipidemia as a likely mechanism by which hypertension occurs during 3 weeks of a HFD.

In conclusion, our findings demonstrate plasma insulin is a likely mechanism by which rapid increases in MAP occur over the first few days of consumption of a HFD. In addition, dyslipidaemia does not appear to develop after 3 weeks of fat feeding suggesting plasma lipid profiles do not play a role in the genesis of hypertension in our animal model but may contribute to the development of comorbidities associated with obesity at a later stage.

### Conflict of interest statement

The authors declare that the research was conducted in the absence of any commercial or financial relationships that could be construed as a potential conflict of interest.
